# Unravelling the Complex Relationship between Diet and Nephrolithiasis: The Role of Nutrigenomics and Nutrigenetics

**DOI:** 10.3390/nu14234961

**Published:** 2022-11-23

**Authors:** Viola D’Ambrosio, Pietro Manuel Ferraro, Gianmarco Lombardi, Simonetta Friso, Giovanni Gambaro

**Affiliations:** 1 Department of Translational Medicine and Surgery, Catholic University of the Sacred Heart, 00168 Rome, Italy; 2 U.O.C. Nephrology, Department of Medical and Surgical Sciences, A. Gemelli University Hospital Foundation IRCCS, 00168 Rome, Italy; 3 Division of Nephrology, Department of Medicine, University of Verona, 37126 Verona, Italy; 4 Unit of Internal Medicine, Department of Medicine, University of Verona, 37126 Verona, Italy

**Keywords:** kidney stones, diet, nutrigenomics, epigenomics

## Abstract

Nephrolithiasis is an increasingly prevalent condition, especially in high income countries, and is associated with high morbidity. Extraordinary progress in genetics made the identification of genetic forms of nephrolithiasis possible. These genetic diseases are usually rare and do not account for the most common forms of nephrolithiasis that are the result of several factors such as environment, dietary habits, and predisposing genes. This knowledge has shaped what we classify as nephrolithiasis, a condition that is now recognized as systemic. How and to what extent all these factors interact with one another and end in kidney stone formation, growth, and recurrence is not completely understood. Two new research fields have recently been trying to give some answers: nutrigenomics and nutrigenetics. These fields have the aim of understanding the intricate diet/genome interface that influences gene expression regulation mainly through epigenetic mechanisms and results in specific medical conditions such as cancer, metabolic syndrome, and cardiovascular diseases. Epigenetics seems to play a crucial role and could represent the link between environmental factors, that we are constantly exposed to, and risk factors for nephrolithiasis. In this systematic review, we summarize all the available evidence of proven or hypothesized epigenetic mechanisms related to nephrolithiasis.

## 1. Introduction

Nephrolithiasis is a common condition whose prevalence has been increasing in recent decades [1]. Calcium-containing kidney stones are the most common type and account for almost 75% of the cases [2]. This condition is generally referred to as idiopathic stone disease and is different from the much rarer secondary calcium nephrolithiasis (caused by monogenic disorders, hyperparathyroidism, and other endocrine disorders, such as malabsorption). This review specifically addresses idiopathic kidney stones, which are hereinafter referred to as kidney stones or nephrolithiasis. The increasing prevalence and studies on the heritability of kidney stones have paved the way for our understanding that nephrolithiasis results from a complex interaction of predisposing genes and environmental factors [3,4]. This hypothesis has been reinforced by evidence of the association between highly prevalent diseases, such as diabetes and metabolic syndrome, or lifestyle and dietary habits, and kidney stones [5,6,7,8,9]. Diet plays a major role in the incidence of nephrolithiasis. Over the last few decades, several dietary patterns have been associated with an increased risk of nephrolithiasis. In particular, the Western-style diet (i.e., a diet high in fat, calories, and animal protein, and low in fibre and plant-based proteins) has been blamed for the increased incidence of kidney stones in Japan [8]. Although not fully understood, the underlying causes of this association have been investigated, linking lithogenic dietary patterns with risk factors for nephrolithiasis, such as reduced urinary excretion of citrate and magnesium, increased excretion of calcium, oxalate and sodium, and acidic urinary pH [10,11,12]. The paradigm to explain the relationship between nutrients and nephrolithiasis is mechanistic; nutritional modifications have a direct and immediate impact on urine composition: higher sodium intake leads to higher natriuresis and to higher urinary excretion of calcium, higher oxalate intake (after balancing in the gut with the calcium intake) leads to increased oxaluria, and higher animal protein/sulphated amino acid intake leads to a bodily acidogenic tide compensated by more acidic urine. The beneficial effect on the development of kidney stones of dietary patterns, such as the Dietary Approach to Stop Hypertension (DASH) diet [12] or the Mediterranean diet [13,14], is explained in the mechanistic way of the previous paradigm [15]. In fact, both diets are characterised by a high intake of fruits and vegetables (a diet rich in alkali) and a reduced intake of animal protein (a diet poor in acids) and sodium. However, there are nutrition-dependent conditions that are associated with the risk of developing nephrolithiasis, such as insulin resistance, diabetes, obesity, and metabolic syndrome, and whose association cannot be entirely mechanistically explained [13].

A large body of evidence now supports the idea that nephrolithiasis is one of the manifestations of systemic conditions, which include bone and cardiovascular (CV) diseases [14,16,17]. While mechanistic explanations might be possible for the association between metabolic bone disease and kidney stones, the mechanisms of the association with CV morbidities are not straightforward [13].

Several studies have demonstrated that bone remodelling and atherosclerotic processes are epigenetically regulated [18]. Whether and to what extent the pathogenesis of nephrolithiasis and its systemic nature are mediated by epigenetic mechanisms remains unknown. Many environmental factors interact with genes through epigenetic mechanisms in human diseases, and nutrition is one of these factors [19]. Several studies have addressed different dietary components that can modify epigenetic processes, thereby modulating gene expression and metabolic effects [20].

Nutrigenomics is the science that studies the influences of micro- and macronutrients and dietary patterns on gene expression; nutrigenetics studies the role of genetic polymorphisms (e.g., single nucleotide polymorphisms; SNPs) on the relationship between dietary patterns and phenotypes. In this systematic review, we aim to summarise recent evidence on the role of nutrigenomics in nephrolithiasis, with a focus on potential epigenetic mechanisms.

## 2. Materials and Methods

In this narrative review, we searched the available literature about nutrigenomics, nutrigenetics, epigenetics, and nephrolithiasis and its risk factors/correlates. We selected papers from PubMed using the following search: (“epigen*”[Title/Abstract] or nutriepigen*[Title/Abstract]) and (“nutri*”[Title/Abstract] or “diet*”[Title/Abstract]). This first search was then coupled with risk factors/correlates of nephrolithiasis and (nephrolithiasis[Title/Abstract] or “kidney stones”[Title/Abstract] or nephrolithiasis[mesh]); and calcium[Title/Abstract]; and phosphorus[Title/Abstract]; and magnesium[Title/Abstract]; and acid-base[Title/Abstract]; and supersaturat*[Title/Abstract]; and vitamin D[Title/Abstract].

All the papers were included irrespective of the language, type of article, or year of publication. We included papers that reported at least one of the following:

1. For any given nutrient/dietary pattern, an epigenetic mechanism on either kidney stone formation or risk factors/correlates of nephrolithiasis
2. For a nutrient/dietary pattern already known to be associated with kidney stones or risk factors/correlates of nephrolithiasis, an epigenetic mechanism in general (not necessarily related to kidney stone formation)

A total of 120 references were read in full and their main findings are summarised in this review.

## 3. Epigenetics in Nephrolithiasis

The term ‘epigenetics’ literally means ‘above the genome’ and is defined as any process that alters gene expression without altering the DNA sequence. Epigenetic phenomena are stable and heritable and are of fundamental importance in regulating gene expression. To date, several epigenetic mechanisms have been identified in eukaryotic cells, among which DNA methylation (usually associated with gene silencing when the cytosine-guanine dinucleotide sequence-rich regions [CpG islands] of gene promoters are methylated), histone modifications (which allow the switch from euchromatin to heterochromatin and vice versa and therefore regulate gene expression; this includes histone acetylation, methylation, phosphorylation, and ubiquitination), noncoding RNA (ncRNA)-associated gene silencing (ncRNAs include microRNAs [miRNAs], short interfering RNAs [siRNAs], and long noncoding RNAs [lncRNAs]). Given the ability to regulate gene expression, epigenetic modifications may affect multiple cellular and metabolic pathways and have been considered in the pathophysiology of many diseases, especially environment-associated diseases. It is therefore not surprising that epigenetics represents a link between medical conditions and nutrition, an environmental factor to which we are constantly exposed [21]. Lifestyle, environmental factors, and diet have been proven to affect and regulate the genome via epigenetic modulations, making it clear that highly prevalent diseases, such as CV diseases, diabetes, and metabolic syndrome, are the result of intricate biological mechanisms that interact with one another [22,23,24]. Nephrolithiasis is among the diseases with increasing prevalence, especially in high-income countries. Although nephrolithiasis is a complex multisystemic condition that depends on both genetic predisposition and environmental factors, many aspects of this disease, especially in idiopathic stone formers, are not completely understood. Unresolved issues, such as intra- and interindividual variability or the recurrence of kidney stones in the absence of specific triggers, suggest that there are other factors involved in its pathogenesis [25]. Among these potential factors, epigenetic modifications may unravel the link between genetic predisposition and environmental factors that are associated with nephrolithiasis. Recently, Khatami et al. demonstrated epigenetic differences between recurrent kidney stone formers and healthy subjects [26]. More specifically, their aim was to quantitatively assess promoter methylation in three genes that are frequently identified in recurrent kidney stone formers and whose expression patterns are controlled by promoter methylation: vitamin D receptor (VDR), calcium-sensing receptor (CaSR), and claudin 14 (CLDN14). Promoter methylation of the three genes was significantly different between recurrent stone formers and controls when methylation status was classified as unmethylated (<9%), methylated (9–29%), or highly/hypermethylated (>29%). However, when methylation status was classified only into unmethylated (<9%) and methylated (≥9%), a statistically significant difference was observed only for the VDR promoter and the distal promoter region of the CLDN14 gene, leading to the hypothesis that epigenetic changes of these two genes’ expression patterns may be of fundamental importance in recurrent kidney stone formation. Both VDR and CLDN14 regulate the urinary excretion of calcium. VDR is expressed by many cell types in the kidney; however, it is highly expressed in proximal and distal tubule cells, where it regulates the expression of genes, such as calbindins and CaSR. High levels of VDR upregulate calbindins and CaSR expression, leading to high urine calcium excretion and a consequent increased risk of stone formation [27]. Claudins play a major role in kidney tubular handling of calcium. They are transmembrane proteins of the epithelial tight junctions that control the transport of calcium and other divalent ions through the epithelium. Data from Khatami et al. suggested that promoter hypermethylation may cause decreased expression of CLDN14, an interesting finding as mutations in the genes encoding for some claudins may cause high urine calcium excretion [28,29,30,31]. Moreover, CLDN14 inhibits claudin 16 (CLDN16) and 19 (CLDN19), which are expressed in the tight junctions of the thick ascending limb of the loop of Henle, and whose function is paracellular reabsorption of Ca^2+^ and Mg^2+^ via a pathway mediated by CaSR and other intracellular intermediate factors [32].

## 4. Nutrigenomics

**Figure 1:**
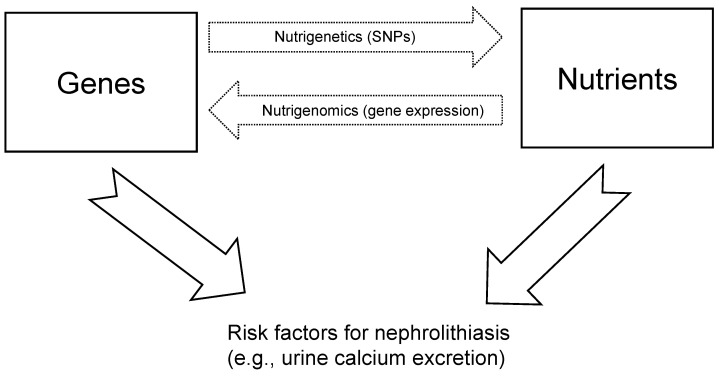
Schematic representation of the link between nutrients, genes, and risk factors for nephrolithiasis.

Nutritional genomics or nutrigenomics is the science that studies the role of nutrients or dietary patterns in gene expression. Nutriepigenomics is therefore the branch of nutrigenomics that studies the epigenetic modifications on gene expression exerted by nutrients and food compounds (Figure 1).

Numerous nutrients involved in epigenetic changes have been identified, comprising methyl donors, amino acids, vitamins, minerals, polyphenols, other phytochemicals, and fatty acids (FAs) [33].

In the following sections, we report the available evidence on nutrigenomics and nephrolithiasis with a focus on epigenetic mechanisms. The relationships between food compounds, dietary patterns, and risk factors/correlates for kidney stone formation and potential underlying epigenetic mechanisms are summarised in Table 1.

## 5. Food Compounds

### 5.1. Acetic Acid (Vinegar)

In a Chinese epidemiological study, the daily intake of vinegar was shown to be associated with a reduced risk of nephrolithiasis [26]. Zhu et al. demonstrated that the administration of vinegar in rat models of calcium oxalate (CaOx) nephrolithiasis reduced urine calcium and increased citrate excretion, thereby reducing the risk of CaOx aggregation and stone formation [34]. The authors demonstrated both in vitro and in vivo that vinegar and acetic acid (the main component of vinegar) act via an acetate-enhanced epigenetic regulatory mechanism by which acetate increases histone H3 acetylation in renal tubular cells and stimulates the expression of miRNAs that suppress the expression of NaDC1 and CLDN14 by enhancing expression of microRNAs -130a-3p, -148b-3p and -374b-5p by increasing H3K9 and H3K27 acetylation at their promoter regions [34].

NaDC1 is a sodium/dicarboxylate cotransporter encoded by the SLC13A2 gene, which is mainly expressed in the proximal tubule and whose function is to reabsorb freely filtered citrate and other Krebs cycle intermediates (e.g., succinate) [38]. Reduced activity of NaDC1 leads to decreased reabsorption of citrate from the tubular lumen, and thus reduces the risk of nephrolithiasis. Furthermore, as previously described, CLDN14 is a tight junction protein that regulates paracellular calcium handling in the thick ascending limb of the loop of Henle. Its downregulation leads to increased reabsorption of Ca^2+^ mediated by CLDN16/19 and a subsequent reduction in calcium urinary excretion. The benefits of vinegar on CaOx kidney stone formation have been recently confirmed by Liu et al. in a CaOx nephrolithiasis rat model [35]. The working hypothesis was that vinegar decreases CaOx crystal formation and adhesion to tubular epithelial cells by regulating gut microbiota and tubular epithelial cell tight junctions. Disruption of the gut microbiota has been associated with CaOx formation through increased absorption and decreased secretion of oxalate in the gut [39,40,41,42,43]; this eventually leads to increased urinary oxalate excretion, supersaturation, and CaOx crystal formation. The disruption of tight junctions induced by CaOx leads to the apoptosis of tubular epithelial cells, increasing the exposure on the luminal surface of crystal-binding molecules, such as hyaluronan, and promoting crystal adhesion to tubular cells [44,45]. Of note, Liu et al. found that dietary administration of vinegar reduced urinary oxalate excretion, CaOx crystal deposition in the kidney, and increased serum acetate (one of the main components of vinegar) concentration [35]. Furthermore, they found that serum acetate increased the expression of occludins in tubular epithelial cell tight junctions both at the messenger RNA (mRNA) and the protein level. The underlying mechanisms have not been fully elucidated; however, one can speculate that, similar to the effects reported by Zhu et al. [25], the effect of vinegar on occludins might be due to epigenetic modifications at a tubular level, as described in other pathological conditions [46,47].

### 5.2. Amino Acids—L-Arginine

Among the results reported by Liu Yu et al., in gut microbiota, a metabolic pathway involved in arginine synthesis was found to be increased in the vinegar group compared with the control group [35]. Clinical or biochemical manifestations of the increased metabolic pathway were not described; however, the effect of dietary supplementation of L-arginine has been previously described in a CaOx stone former and a uninephrectomised rat model [36]. Dietary L-arginine supplementation significantly decreased urinary calcium and increased citrate excretion; interestingly, it also increased urinary sodium and albumin excretion, two well-known risk factors for kidney damage. Urinary oxalate excretion was not evaluated; however, L-arginine-treated rats showed less CaOx crystal deposition in the kidney compared with controls. Additional evidence of the potential benefit of L-arginine dietary supplementation on CaOx stone formation was reported by Pragasam et al., who demonstrated the role of L-arginine in reducing oxalate excretion and oxidative stress-induced kidney injury, thereby reducing the risk of kidney stone formation in a hyperoxaluric rat model [48]. L-arginine is an amino acid found mainly in animal protein (dairy, fish, and poultry) and nuts (walnuts, hazelnuts, pecans, peanuts, almonds, and cashews). Since dietary L-arginine acts via epigenetic mechanisms in other conditions and metabolic pathways [49,50], it is tempting to hypothesise the role of epigenetic mechanisms behind the effects of L-arginine on nephrolithiasis.

### 5.3. Fructose

Fructose is a macronutrient of interest in nephrolithiasis. A diet high in fructose, which can be mainly found in processed food and carbonated beverages, is strongly associated with an increased incidence of nephrolithiasis [37,51]. High fructose intake has previously been associated with high urinary calcium and oxalate excretion in a magnesium-depleted mouse model [52,53] and in healthy individuals [54,55]. Fructose intake also increases insulin resistance, which in turn is associated with a low urinary pH [56] (a risk factor for uric acid kidney stones). The exact mechanisms by which fructose modifies urine composition are still not completely understood and a specific epigenetic mechanism exerted by fructose in nephrolithiasis or risk factors for kidney stone formation have not been described. However, from available data, we know that fructose acts via epigenetic mechanisms (ncRNA and DNA methylation) in other conditions [57,58,59], such as nonalcoholic fatty liver disease (NAFLD) [60,61,62]. Therefore, we can speculate that fructose might act via epigenetic mechanisms to regulate the expression of genes that encode crucial transporters of anti- and prolithogenic urinary molecules.

### 5.4. Sodium Chloride

Sodium chloride is a widely used food compound in our diet. In Western countries, sodium chloride serves as an additional compound to improve the taste of food and is used also as a preservative. This is the reason why diets largely based on processed food, such as the Western diet, are generally high in sodium chloride. High dietary salt intake has been associated with several medical conditions, such as hypertension [63], CV disease [64], and nephrolithiasis [65,66]. The mechanisms underlying these associations have been extensively studied over the last two decades, and the results have highlighted a strong relationship between high dietary sodium chloride intake, increased urinary excretion of sodium, and high urinary calcium excretion [10,67]. Several studies reported a direct correlation between increased dietary sodium chloride intake and high urinary calcium excretion and vice versa (reduced salt intake and decreased urinary calcium excretion) [68,69,70]. Furthermore, the reduction in calciuria caused by the reduced intake of salt might lead, through a homeostatic mechanism, to a reduction in the absorption of calcium in the more proximal portions of the intestine, leaving a greater proportion of it more available at the distal level to form greater quantities of nonabsorbable calcium oxalate [71]. Regarding the underlying physiological mechanisms, Yatabe et al. conducted a study on murine models and found that a high sodium chloride diet was associated with decreased expression of claudin 2 (CLDN2) in the proximal tubule [72]. Interestingly, they also demonstrated increased expression of proteins involved in transcellular calcium reabsorption in the distal convoluted tubule: transient receptor potential cation channel subfamily V member 5 (TRPV5; an apical calcium transporter), calbindin-D_28k_ (an intracellular calcium-binding protein), and sodium-calcium exchanger 1 (NCX1; a basolateral sodium-calcium exchanger). These changes did not seem to compensate for the CLDN2-mediated decrease in calcium reabsorption in the proximal tubule. The mechanisms behind this shift in protein expression in response to sodium load remain unclear; however, it would be of interest to investigate the possible role of epigenetic mechanisms. Amara et al. demonstrated epigenetic alterations and a predisposition to cardiac dysfunction in uninephrectomised rats exposed to a high sodium chloride diet, although a cause-effect mechanism has not yet been elucidated [73].

### 5.5. Vitamin D

In the topic of nutrigenomics in nephrolithiasis, vitamin D has received much of the focus. In the previous section, we reported how epigenetic changes in vitamin D receptor (VDR) were associated with an increased risk of nephrolithiasis [18,19]. Endogenous and dietary vitamin D act via their active metabolite, 1α,25-dihydroxyvitamin D3 (1.25 (OH)_2_-vitamin D), and regulate the genome, epigenome, and transcriptome involved in calcium homeostasis and other physiological or pathological pathways, such as innate and adaptive immune response, osteoporosis, sarcopenia, and cancer [74]. The ability of vitamin D to regulate genome expression has been investigated as a possible cause of interindividual variability in response to exogenous supplementation. The same could be applied to high urinary calcium excretion and the risk of kidney stone formation. Due to the known effect of vitamin D on increasing the intestinal absorption of calcium and phosphorus, its association with nephrolithiasis has been of interest both in research and in clinical practice; however, prospective interventional studies are scarce. In addition to nephrolithiasis, vitamin D is crucial in bone metabolism. Several studies [75] have demonstrated the link between the genome and environment in the development of osteoporosis and vitamin D, and its receptor VDR may act as a mediator. Other studies, both in animal models and in humans, found an association between vitamin D deficiency during pregnancy and the risk of medical conditions in offspring, such as inflammation and adiposity [76], allergies [77], ageing [78], and osteoporosis [79] via epigenetic modifications. In a review by Snegarova et al., hypovitaminosis D was associated with an increased incidence of obesity, insulin resistance, diabetes, and osteoporosis, all conditions that are associated with nephrolithiasis [80]. The vitamin D pathway is tightly regulated by epigenetic mechanisms and seems to also regulate epigenetic modifications. It is therefore interesting to speculate an underlying epigenetic mechanism regulating a possible prolithogenic response to endogenous and exogenous vitamin D. However, direct data to corroborate or refute this hypothesis are currently unavailable.

### 5.6. Calcium

Calcium plays a crucial role in nephrolithiasis. From a biochemical perspective, high urinary excretion of calcium is one of the main risk factors for nephrolithiasis, and CaOx and calcium phosphate are the most common components of kidney stones. The role of dietary calcium has long been debated. Although it was previously believed that a low calcium diet would reduce the risk of kidney stones, it is now widely accepted that the opposite is true; in fact, a diet containing 1000–1200 mg/day of calcium reduces the risk of kidney stone formation by reducing the intestinal absorption of oxalate [81,82]. Moreover, it has been reported that when patients were advised to reduce their dietary intake of milk and dairy, they tended to increase their animal protein intake [83]. It is currently unknown whether dietary calcium per se induces epigenetic changes that would predispose to nephrolithiasis; however, there are reports in the literature of epigenetic mechanisms induced by dietary calcium [84,85,86]. In a paper published by Takaya et al., a low-calcium diet in mice during pregnancy was associated with insulin resistance and reduced insulin secretion in offspring via a heritable epigenetic mechanism [87]. In humans, insulin resistance plays a role in uric acid nephrolithiasis [88]. Therefore, it would be interesting to investigate whether a link exists between dietary calcium, insulin resistance, and uric acid nephrolithiasis in vivo via epigenetic mechanisms. The interaction between DNA polymorphisms and dietary calcium and its impact on the risk of calcium nephrolithiasis is discussed in the ‘nutrigenetics’ section.

### 5.7. Magnesium

Magnesium is an abundant element in our body, although its free circulating concentration makes up only 1% of the total body magnesium; the rest is in combination with calcium and phosphate in bone and soft tissue [89]. It is because of this affinity for calcium and phosphate, two players in kidney stone formation, that magnesium has drawn attention as a potential kidney stone inhibitor. In recent decades, several in vivo and in vitro studies have demonstrated the ability of magnesium to reduce the risk of nephrolithiasis. Magnesium supplementation in kidney stone formers increases magnesium and citrate excretion [90] and decreases oxalate excretion [91]; this could represent an underlying mechanism for a reduction in the incidence of new kidney stone events [68]. Curhan et al. demonstrated an inverse association between urinary magnesium and nephrolithiasis. However, clinical evidence that supports the beneficial effects of magnesium supplementation alone in stone formers is scarce [92,93]. From a physiological point of view, urinary magnesium acts as an inhibitor of crystal formation; it competitively binds oxalate, forming magnesium oxalate, which is more soluble than CaOx [90]. Whether or not magnesium influences the risk of kidney stone formation via epigenetic modifications has not yet been evaluated to our knowledge. However, a subclinical magnesium deficit may stimulate intracellular inflammatory pathways via possible epigenetic mechanisms, leading to chronic inflammation-related diseases, such as type 2 diabetes, metabolic syndrome, osteoporosis, and CV disease [94,95]. These conditions are all associated with an increased incidence of nephrolithiasis. In an animal model of magnesium deficiency-induced disease, inflammatory and apoptosis response-related genes were modulated by epigenetic mechanisms [96]. Therefore, it would be interesting to explore whether magnesium deficiency or low magnesium urinary excretion, in addition to other lifestyle and environmental factors, could cause prolithogenic epigenetic modifications.

### 5.8. Phosphate

Phosphate is an essential molecule for cellular metabolism and bone health. Up to 80–85% of body phosphate is present in the bone as salt, and the remaining 15–20% circulates in the serum as free ion or protein-bound phosphate. Phosphate concentration ultimately depends on dietary intake, gastrointestinal absorption, and urinary excretion; it shifts between the intracellular and the extracellular fluid and is tightly regulated by hormones, such as PTH, FGF23, and vitamin D [97]. Phosphate is freely filtered by the glomerulus and is mainly reabsorbed by the proximal tubule via transcellular Na^+^-driven transport. Tubular phosphate handling has been of interest in nephrolithiasis. Increased tubular phosphate leak increases the risk of urinary supersaturation with calcium phosphate and lowers plasma phosphate concentration. This causes an increased conversion of 1.25 (OH)_2_-vitamin D and the enhanced intestinal absorption of calcium and phosphate by the gut, resulting in high urinary calcium excretion [98,99,100]. In addition to the vitamin D-mediated effects, urinary phosphate leak affects other prolithogenic urinary parameters, such as uric acid, oxalate, citrate, the incidence and recurrence of nephrolithiasis, and bone demineralisation [98]. To our knowledge, the role of phosphate intake in nephrolithiasis has not yet been evaluated. It must be noted that all foods contain phosphate; however, it is the type and bioavailability of the phosphate that regulates intestinal absorption. Processed food is high in inorganic phosphate, which has the highest bioavailability. Animal and plant-based proteins are both high in organic phosphate; however, phosphate from animal proteins has higher bioavailability [101]. The Western diet provides a high phosphate load. Given the link between urinary phosphate and nephrolithiasis [98], and the link between a Western diet and nephrolithiasis [10], it would be interesting to explore whether and how dietary phosphate intake is associated with prolithogenic risk factors and whether this link is mediated by epigenetic mechanisms. Dietary phosphate intake has been associated with altered miRNA expression in neck squamous cell carcinoma [102], indicating that this element is potentially able to activate epigenetic pathways.

### 5.9. Dietary Acid-Base

One of the main functions of the kidney is to maintain acid-base balance and serum pH in the physiological range (7.35–7.45). Many factors have been demonstrated to affect this equilibrium; among them, diet plays an important role. Previous studies comparing high acid-load diets (e.g., the Western diet) to alkalinising diets (e.g., vegan or vegetarian diets) have demonstrated beneficial effects on general health outcomes, and more specifically kidney outcomes [4]. A high dietary acid load can imbalance acid-base homeostasis, leading to physiological responses in the kidneys and bone to buffer acidity. These responses include increased reabsorption of citrate by the renal proximal tubule and increased reabsorption of bone by osteoclasts to release calcium salts that buffer serum acids at the expense of increased excretion of calcium in the urine. Diet-induced acidosis stimulates osteoclast activity in bone reabsorption through receptor activation of the NF-κB ligand (RANKL) pathway [103]. Low urine citrate, high urine calcium, and acidic urine are risk factors for kidney stone formation, and the association between diets with an increased acid load and nephrolithiasis has been extensively studied. Whether other mechanisms, such as epigenetic modifications, play a role in this relationship has not been evaluated. However, dietary acid load, even when transient, may upregulate biological pathways, such as the cortisol pathway, as demonstrated by Espino et al. [104], or insulin growth factor 1 (IGF-1) [105]; however, the data in the literature is controversial.

## 6. Dietary Patterns

### Western Diet

After analysing the association between single-food compounds or elements and nutrigenomics, it is natural to question whether dietary patterns might predispose to certain clinical conditions via epigenetic mechanisms. Dietary patterns and habits have been of key interest in nephrolithiasis, especially in high-income countries where food processing is associated with highly prevalent diseases, including hypertension, CV disease, obesity, insulin resistance, and diabetes. As previously mentioned, the Western diet involves high animal protein intake, low fruit and vegetable intake, and sweetened beverages [51]. Several studies have been conducted comparing different dietary patterns on the risk of nephrolithiasis [106,107]. It is currently accepted that plant-based diets are high in potassium, magnesium, and alkalinising agents, such as citrate, and thus act as a protective factor against nephrolithiasis. These diets include the vegan or vegetarian diet, the DASH diet [108], and the Mediterranean diet [109]. Of particular interest is the role of potassium in kidney stone formation. Previous studies have demonstrated a strong inverse relationship between dietary potassium and the incidence of nephrolithiasis; however, the relationship was even stronger when the dietary animal protein to potassium ratio was considered [110]. This underlies the importance of a potassium source and accompanying anion (chloride vs. citrate), with a net beneficial effect of potassium citrate that can be mainly found in fruits and vegetables, even with regard to urinary excretion of calcium [111]. Although a protective effect of urinary potassium excretion has been demonstrated for the progression of CKD [112] and hypertension [113], whether this is true for kidney stone formation and whether this could be mediated by epigenetic mechanisms has yet to be consistently proven.

Regarding dietary patterns and epigenetic mechanisms, Choi et al. conducted a study in murine models and demonstrated that the Western diet reduced DNA methylation [114]. Reductions in DNA methylation have been observed in other physiological processes, such as ageing. This study demonstrated that dietary patterns could indeed induce epigenetic modifications, and it would be interesting to explore a possible epigenetic link between different types of diet and nephrolithiasis.

## 7. Nutrition-Induced Epigenetic Modifications as Determinants of the Multisystemic Nature of Nephrolithiasis and the Possible Role of Fatty Acids

Epigenetic modifications may influence many cellular and metabolic pathways relevant to the expression of carriers at the level of the tubular and intestinal epithelium, the bone in the interface between osteoclasts and osteoblasts [18], and in the vessel wall. Epigenetic modifications regulate the expression of several genes in different tissues and have an impact on the pathophysiology of many diseases, especially the ones that are associated with the environment [115,116]. Some cellular and metabolic pathways are shared by different tissues (e.g., Wnt cascade, NF-κB cascade, and macrophage colony-stimulating factor). Therefore, epigenetic modifications induced by certain environmental conditions, namely nutrition, may simultaneously affect the biology of different tissues and organs. To our knowledge, the systemic impact (i.e., the simultaneous effect on different organs/tissues) of nutrigenetics has never been investigated apart from studies on the epigenetic regulation of fetal development and growth and the long-term pathological effects in adulthood of mother’s conditions during pregnancy [117]. We speculate that this approach might be fruitful to explain the systemic nature of nephrolithiasis. In this respect, it is possible to revisit and partly reinterpret previous data on the association of nephrolithiasis with a derangement of FAs.

FAs may also be an interesting player in nutrigenomics. FAs, such as α-linolenic acid, eicosapentaenoic acid (EPA), and docosahexaenoic acid (DHA), modify DNA methylation sites in several genes associated with CV morbidities [118]. Interestingly, *n* − 3 polyunsaturated FAs (i.e., EPA, DHA) have been associated with the prevention of CV disease, metabolic alterations (i.e., lipid metabolism disturbances), and other chronic diseases (obesity, type 2 diabetes mellitus, and NAFLD) [118]. Abnormal FA 6-desaturase activity has been reported in nephrolithiasis and is responsible for the significantly higher content of arachidonic acid in lipoproteins and erythrocyte membrane phospholipids, lower content of linoleic acid, and a higher arachidonic/linoleic acid ratio compared with healthy subjects [119]. FA 6-desaturase, the rate-limiting enzyme in the biosynthetic pathway of highly unsaturated FAs, is modulated by nutritional factors, specifically protein and glucose intake, and dietary lipid composition [120]. Therefore, we may propose a scenario in which nutritional factors, by modulating the composition of FAs in plasma and cell membrane phospholipids, namely the *n* − 6/*n* − 3 ratio, can exert epigenetic effects on several genes and metabolic pathways relevant to the pathogenesis of nephrolithiasis, atherosclerosis, CV disease, and bone. The experimental manipulation of FA 6-desaturase activity and/or nutritional supplementation with *n* − 3 FA (fish oil) that altered plasma and cell membrane phospholipid composition was associated with changes in biomarkers of bone turnover [121,122,123].

## 8. Conclusions

A deeper understanding of the pathophysiological mechanisms underlying nephrolithiasis is of crucial importance in the diagnosis, treatment, and prevention of this increasingly prevalent condition. Retrospective and prospective association studies made it clear that nephrolithiasis is a systemic condition, and several factors, such as the environment, dietary habits, and predisposing genes, all play a role in its development.

Although it is possible to speculate that epigenetic mechanisms might also play a role in the formation of noncalcium-containing stone types, especially those that are sensitive to urinary pH such as uric acid, cystine, or calcium phosphate, no evidence is available in the literature. Nutrigenomics may represent a crucial tool for pursuing personalised treatment, and unravelling underlying epigenetic mechanisms may lead to the discovery of therapeutic targets and the development of nutraceutical drugs with an impact on stone formation and its systemic manifestations.

## Figures and Tables

**Table 1:** Schematic summary of the relationship between food compounds and pro or antilithogenic urinary phenotypes mediated by proved or hypothesized epigenetic mechanisms.

Food	Epigenetic Mechanism	Phenotype
Acetic acid (vinegar) [34]	ncRNA-mediated gene silencing (miRNA) by promoting expression of microRNAs -130a-3p, -148b-3p, and -374b-5p by increasing H3K9, H3K27 acetylation at their promoter regions	Reduced urinary calcium Increased urinary citrate
Acetic acid (vinegar) [35]	unknown	Reduced urinary oxalate Reduced CaOx crystals adhesion Reduced plasma acetate -> increased occludins in tight junctions -> reduced CaOx stone formation
L-arginine [36]	unknown	Reduced urinary calcium Increased urinary citrate
Fructose [37]	unknown	Increased urinary calcium Increased urinary oxalate
